# Short-term duodenal seal oil administration normalised n-6 to n-3 fatty acid ratio in rectal mucosa and ameliorated bodily pain in patients with inflammatory bowel disease

**DOI:** 10.1186/1476-511X-5-6

**Published:** 2006-03-20

**Authors:** Tormod Bjørkkjær, Johan G Brun, Merete Valen, Gülen Arslan, Ragna Lind, Linn A Brunborg, Arnold Berstad, Livar Frøyland

**Affiliations:** 1 National Institute of Nutrition and Seafood Research (NIFES), Bergen, Norway; 2 Department of Biomedicine, University of Bergen, Bergen, Norway; 3 Division of Rheumatology, Institute of Medicine, Haukeland University Hospital, Bergen, Norway; 4 Division of Gastroenterology, Institute of Medicine, Haukeland University Hospital, Bergen, Norway

## Abstract

**Background:**

A high dietary intake of n-6 compared to n-3 fatty acids (FAs) may promote the production of pro-inflammatory eicosanoids and cytokines. In two recent studies, short-term (10-day) duodenal administration of n-3 polyunsaturated fatty acid rich seal oil ameliorated joint pain in patients with inflammatory bowel disease (IBD). Using unpublished data from these two studies we here investigated whether normalisation of the n-6 to n-3 FA ratio in blood and tissues by seal oil administration was associated with improved health related quality of life (HRQOL) as assessed by the generic short-form 36 (SF-36) questionnaire.

**Results:**

In the first pilot study, baseline n-6 to n-3 FA ratio in rectal mucosal biopsies from 10 patients with IBD (9 of those had joint pain) was significantly increased compared with that in 10 control patients without IBD or joint pain. Following seal oil administration, the n-6 to n-3 FA ratio of the IBD-patients was significantly lowered to the level seen in untreated controls. In the subsequent, randomized controlled study (*n *= 19), seal oil administration reduced the n-6 to n-3 FA ratio in blood similarly and also the SF-36 assessed bodily pain, while n-6 FA rich soy oil administration had no such effect.

**Conclusion:**

In these two separate studies, short-term duodenal administration of seal oil normalised the n-6 to n-3 FA ratio in rectal mucosa and improved the bodily pain dimension of HRQOL of patients with IBD-related joint pain. The possibility of a causal relationship between n-6 to n-3 FA ratio in rectal mucosa and bodily pain in IBD-patients warrants further investigations.

## Background

Most of our dietary fat is derived from vegetable oils, particularly soy oil, which is rich in the n-6 fatty acid (FA) linoleic acid (18:2n-6, LA). LA is the precursor of arachidonic acid (20:4n-6, AA). Especially linseed oil, and to a less extent rapeseed oil and soy oil contain substantial amounts of the n-3 FA α-linolenic acid (18:3n-3, ALA). However, in humans the *in vivo *conversion of ALA to the n-3 polyunsaturated fatty acids (PUFAs) eicosapentaenoic acid (20:5n-3, EPA), and particularly docosahexaenoic acid (22:6n-3, DHA), is limited [1]. Thus, fatty fish and n-3 PUFA supplements are important sources of the 'marine' n-3 PUFAs EPA and DHA [2]. These long chain n-3 PUFAs decrease the production of pro-inflammatory eicosanoids and cytokines, directly by replacing AA in blood and tissues and inhibiting AA metabolism or indirectly by altering gene transcription [3]. The current Western diet with excessive n-6 FAs compared with n-3 FAs yields a high ratio of n-6 to n-3 FAs, especially AA to EPA, e.g. in the cell membrane phospholipids. This may promote chronic inflammatory diseases like inflammatory bowel disease (IBD) and rheumatic disorders [4-6].

IBD includes Crohn's disease (CD) and ulcerative colitis (UC), both chronic inflammatory diseases of the gastrointestinal tract, causing abdominal pain and bloody diarrhoea. Musculoskeletal (rheumatic) complications, including joint pain with or without objective signs of arthritis, are common extraintestinal manifestations [7-9], contributing to the generally poor health related quality of life (HRQOL) of IBD-patients [10]. Also, non-steroidal anti-inflammatory drugs (NSAIDs) are poorly tolerated by these patients. IBD-patients with joint pain therefore often call for a safe and effective alternative treatment. In a short-term (10-day) pilot study we found a beneficial effect on IBD-related joint pain of duodenally administrated n-3 PUFA rich seal oil (10 ml three times daily) [11]. The promising results were supported in a recent randomized controlled study comparing the effects of seal oil and soy oil on IBD-related joint pain [12]. The joint pain ameliorating effect of seal oil persisted for several months after the 10-day treatment period, while soy oil had no such effect.

Using unpublished data from these two studies [11,12] we here investigated whether normalisation of the n-6 to n-3 FA ratio in blood and tissues by seal oil administration was associated with improved HRQOL as assessed by the generic short-form 36 (SF-36) questionnaire.

## Methods

### Patients and controls

In the pilot study, 10 patients with IBD (9 of those had joint pain) went through a gut lavage fluid procedure before and after seal oil treatment [11]. Immediately after emptying their bowels, sigmoidoscopy was performed with a videoendoscope. Mucosal biopsies were taken from the rectum, 20–30 cm above the anus, collected on ice and stored at -80°Celsius. The control patients consisted of 10 males (range 50–67 years, mean 67 years) with prostate cancer, but without IBD or joint pain, routinely assessed before radiation therapy. Mucosal biopsies were taken without prior emptying of the bowels, by rectoscopy from the posterior rectal wall, 10 cm from the anal verge, collected on ice and stored at -80°Celsius. Fatty acid composition in biopsies was analysed by gas liquid chromatography as previously described [11]. In the controlled study, seventeen of the nineteen patients with IBD-related joint pain [12] filled in a translated and validated Norwegian version of the Medical Outcome Study (MOS) SF-36 health survey questionnaire [13-15] before and after seal oil (*n *= 9) or soy oil (*n *= 8) administration, in addition to 1, 2, 4 and 6 months post-treatment. SF-36 is a generic self-administrated HRQOL questionnaire consisting of 36 questions, assessing eight health concepts (physical functioning, role limitations due to physical problems, social functioning, bodily pain, general mental health, role limitations due to emotional problems, vitality and general health perceptions). Final values from the SF-36 questionnaire ranged from 0 (very poor) to 100 (very well). The regional committee for medical research ethics approved these studies and all patients gave written informed consent before inclusion.

### Statistics

Data were analysed and displayed using the GraphPad Prism 4 (GraphPad Software Inc, San Diego, USA) statistical software package or SPSS Release 9.0.0 software (SPSS Inc, Chicago, IL, USA). All values were expressed as mean ± standard error of the mean (SEM). Effect of treatment was calculated as change (in absolute values) from baseline. Area under the curve (AUC, area between the curve and baseline, zero) for the entire period from start of treatment until 6 months post-treatment was calculated using the trapezoid method. Group differences were compared by unpaired Student's *t *test (two-sided). Differences from baseline to end of treatment were evaluated by paired *t*-test. *P *values < 0.05 were regarded as statistically significant.

## Results

### Biopsy fatty acid composition

**Figure 1:**
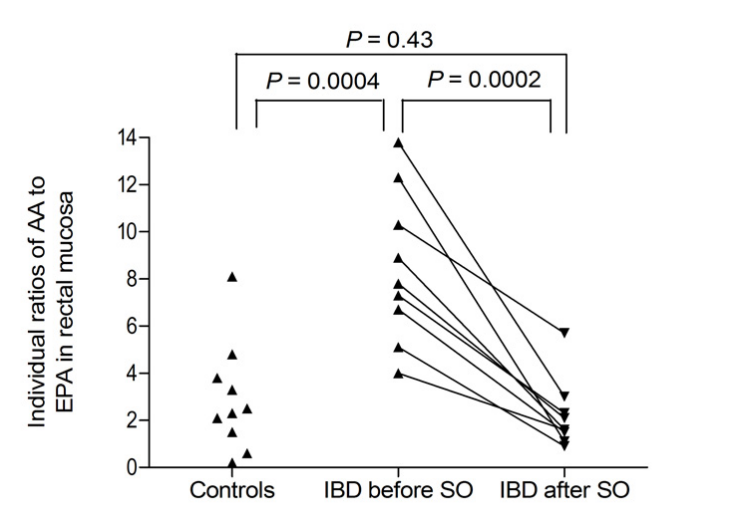
**Biopsy AA to EPA ratio in IBD-patients before and after seal oil compared with controls**. Normalisation of individual ratios of arachidonic acid (20:4n-6, AA) to eicosapentaenoic acid (20:5n-3, EPA) in rectal mucosal biopsies from nine of the ten IBD-patients (data from the pilot study [11]) after seal oil (SO) treatment compared with rectal mucosal biopsies from ten controls.

Compared with controls, higher ratios of n-6 to n-3 FAs (*P *= 0.001) and AA to EPA (8.5 ± 1.1 vs 2.9 ± 0.7, *P *= 0.0004) were found in baseline rectal mucosa from the patients with IBD-related joint pain (Table 1, Figure 1). These differences vanished after seal oil treatment (Table 1, Figure 1). Also a higher Σ n-6 FAs (*P *= 0.04), LA (*P *= 0.02) and 18:1n-9 (*P *= 0.004) and lower Σ n-3 FAs (*P *= 0.0004), EPA (*P *= 0.0003), DHA (*P *= 0.0006), Σ saturated (*P *= 0.01) and 17:0 (*P *= 0.02) were found in rectal mucosa from the patients with IBD-related joint pain before treatment with seal oil (Table 1). These differences also vanished after seal oil treatment (Table1). Seal oil treatment reduced Σ monoenes in rectal mucosa from the IBD-patients to a significantly lower level (*P *= 0.003) than that seen in untreated controls.

**Table 1:** Biopsy fatty acid-profiles from IBD-patients before and after seal oil treatment compared with controls

Fatty acid	Controls	IBD before	IBD after	*P*-value before	*P*-value after
Σ Saturated	36.9 ± 2.1	32 ± 1	31 ± 3	0.01	n.s.
14:0	2.9 ± 0.6	1.4 ± 0.3	1.2 ± 0.3	0.02	0.01
16:0	20.2 ± 0.4	17.9 ± 0.9	17 ± 1	0.008	0.02
17:0	0.90 ± 0.01	0.61 ± 0.08	0.7 ± 0.1	0.02	n.s.
18:0	11.1 ± 0.7	11 ± 1	12 ± 1	n.s.	n.s.
Σ Monoenes	31 ± 4	31 ± 2	24 ± 2	n.s.	0.003
16:1 n-7	1.9 ± 0.2	2.0 ± 0.5	1.5 ± 0.3	n.s.	n.s.
16:1 n-9	0.50 ± 0.01	0.40 ± 0.04	0.37 ± 0.05	n.s.	n.s.
18:1 n-7	2.3 ± 0.1	2.4 ± 0.2	2.0 ± 0.2	n.s.	n.s.
18:1 n-9	19 ± 1	25 ± 1	19 ± 1	0.004	n.s.
20:1 n-9	2.9 ± 0.6	0.13 ± 0.07	0.4 ± 0.3	0.0008	0.003
Σ n-6	17 ± 2	24 ± 2	19 ± 1	0.04	n.s.
18:2 n-6	11 ± 2	16 ± 1	12.1 ± 0.6	0.02	n.s.
20:3 n-6	tr.	1.5 ± 0.2	1.0 ± 0.1	< 0.0001	0.0004
20:4 n-6	5.7 ± 0.9	6 ± 1	6 ± 1	n.s.	n.s.
Σ n-3	9 ± 1	4.1 ± 0.5	9 ± 1	0.0004	n.s.
18:3 n-3	0.5 ± 0.1	0.4 ± 0.1	0.24 ± 0.08	n.s.	n.s.
20:5 n-3	2.6 ± 0.3	0.81 ± 0.05	3.4 ± 0.4	0.0003	n.s.
22:5 n-3	0.7 ± 0.1	0.6 ± 0.2	0.9 ± 0.2	n.s.	n.s.
22:6 n-3	4.6 ± 0.4	2.2 ± 0.3	4.3 ± 0.6	0.0006	n.s.
n-6/n-3	2.2 ± 0.4	6.6 ± 1.1	2.2 ± 0.2	0.001	n.s.

Data from the pilot study [11], IBD-group (*n *= 10) and control group (*n *= 10). Data presented as percentage of fatty acids, mean ± SEM. Identified fatty acids ranged from 94–98 %. tr. = trace. n.s. = not significant (p > 0.05). *P*-value before = controls versus IBD-patients before seal oil treatment. *P*-value after = controls versus IBD-patients after seal oil treatment.

### Health related quality of life

Compared with soy oil, seal oil administration significantly reduced the SF-36 assessed bodily pain (Table 2). SF-36 also revealed significant within group improvements in bodily pain (*P *= 0.004), mental health (*P *= 0.03) and physical component score (*P *= 0.002), and a significant aggravation in emotional role functioning (*P *= 0.02) after seal oil treatment (Table 2). No significant group differences in AUC for the period from before treatment until 6 months post-treatment were found. However significant with-in group improvements were found in AUC for the period from before treatment until 6 months post-treatment; in the seal oil group for bodily pain (*P *= 0.04), mental health (*P *= 0.0009) and mental component score (*P *= 0.02) and in the soy oil group for mental health (*P *= 0.049). Generally both groups improved in the physical dimensions of SF-36 after oil administration, with more profound effects in the seal oil group. Also, there were no changes in the psychometric dimensions of SF-36, except for an aggravation in role limitations due to emotional problems during and soon after oil treatments, but not during follow-up (data not shown).

**Table 2:** SF-36 scores before and after treatment with seal oil or soy oil in IBD-patients

	Before seal oil	After seal oil	Before soy oil	After soy oil	*P*-value
Physical functioning	72.8 ± 6.0	80.0 ± 3.5	68.8 ± 8.3	70.4 ± 9.7	0.36
Role physical	30.6 ± 12.3	34.3 ± 12.5	25.0 ± 14.2	31.3 ± 15.5	0.80
Role emotional	66.7 ± 14.1	41.7 ± 15.1	54.2 ± 17.7	45.8 ± 16.6	0.24
Bodily pain	36.3 ± 6.4	57.7 ± 8.5	35.9 ± 6.3	46.7 ± 10.4	**0.01**
Social functioning	51.4 ± 3.3	51.4 ± 2.5	54.7 ± 2.3	50.0 ± 2.4	0.36
Mental health	67.1 ± 7.1	73.8 ± 6.2	63.0 ± 8.1	65 ± 6	0.31
Vitality	30.0 ± 3.1	38.3 ± 5.7	41.9 ± 8.2	43.8 ± 9.2	0.35
General health	43.3 ± 6.1	51.2 ± 4.9	40.4 ± 5.4	43.6 ± 5.5	0.50
Physical component score	36.7 ± 2.0	42.8 ± 1.9	34.6 ± 2.9	37.0 ± 3.6	0.15
Mental component score	39.5 ± 2.7	37.0 ± 3.5	38.1 ± 4.1	36.7 ± 3.4	0.63

Data from the controlled study [12], seal oil group (*n *= 9) and soy oil group (*n *= 8). No significant group differences at baseline. Boldface numbers = significantly group difference after treatment.

## Discussion

### Fatty acid composition

During the last 50 to 100 years the typical Western diet has contained increasing amounts of n-6 FAs and decreasing amounts of n-3 FAs [6]. Vegetable oils containing much n-6 FAs, especially soy oil, are widely used in foodstuffs and as spreads, mainly due to its low cost. In an evolutionary aspect our ancestors evolved on a diet with a n-6 to n-3 FA ratio close to 1:1, while today this ratio is probably 5–15:1 [6].

The importance of the n-6 to n-3 FA ratio for development of disease was demonstrated in a recent transgenic mouse model [16]. In this study an approximately 1:1 ratio of n-6 to n-3 FAs was achieved in different cells and tissues of *fat-1 *mice compared with a ratio of 20–50: 1 in wild type mice. This protected against cancer growth, cardiac arrhythmia and had most probably collateral health benefits in *fat-1 *mice compared with wild type mice. Similarly, a seal oil-induced lowering of n-6 to n-3 FA ratio, especially AA to EPA, may have health benefits in humans. E.g. by reducing the production of highly pro-inflammatory eicosanoids and cytokines both locally in the intestine and systemically, and possibly thereby also influence joint pain. However, whether amelioration of bodily pain was causally related to the normalisation of the n-6 to n-3 FA ratio in rectal mucosa is not known.

A possible limitation of our biopsy FA comparison was the use of elderly men with prostate cancer as a control group. Elderly people normally eat more n-3 PUFA rich fish than younger people and may have a lower blood and tissue ratio of n-6 to n-3 FAs in general, however we did not assess the diet in this study.

### Health related quality of life

For assessing subjective aspects of a patients' health, i.e. HRQOL, it is important to use a generic HRQOL questionnaire, e.g. the widely used SF-36, together with a disease specific questionnaire as used in our former studies [10-12]. HRQOL is particularly important when objective clinical findings are scarce as often is the case in IBD-related joint pain. In a recent population based cohort study, sixteen percent of 521 IBD-patients had non-inflammatory joint pain with a considerable negative impact on HRQOL [17]. The even more pronounced reduction of HRQOL in our patients might be due to the fact that 10 of our 19 IBD-patients had objective arthritis [12].

The aggravation in role limitations due to emotional problems after both oil treatments may be attributed to anxiety using the nasoduodenal feeding tube, which in some patients resulted in emotional problems and work-related inconvenience.

A limitation with using SF-36 questionnaire in our study was that the SF-36 is designed to assess health status over the last 4 weeks. We used the SF-36 questionnaire just before and after oil administration (10 days) and then at 1, 2, 4 and 6 months post-treatment. Thus we may have lost a further positive (for physical dimensions) or negative (especially for role limitations due to emotional problems) treatment effect when we applied the SF-36 with only a 10-day interval.

### Seal oil versus fish oil

Traditionally, cod liver oil or other fish oils have been used in clinical studies of n-3 PUFAs. However, the initial studies of n-3 PUFAs were performed in Greenland Eskimos known to eat substantial amounts of sea mammals, i.e. seal and whale meat and blubber [18,19]. Greenland Eskimos had a low prevalence of common westernized diseases like myocardial infarction, diabetes mellitus, bronchial asthma, multiple sclerosis and psoriasis [20], notably before the Western diet became more common in the larger communities of Greenland.

Seal oil contains slightly less EPA and DHA than fish oil, but approximately 3 to 4 fold more DPA, giving an equivalent total n-3 fatty acid level [21]. The n-3 PUFAs in fish oil are mainly located in *sn*-2 position of the triacylglycerol (TAG) molecule, while they are located almost exclusively in *sn*-1 or *sn*-3 position of TAG from seal oil [22,23]. Chylomicrons resulting from TAG digestion are designated for lymphatic transport [24]. They reflect the dietary TAGs with respect to both fatty acid profile and molecular position of the n-3 PUFAs [23,25]. However, in a recent review focusing mainly on metabolism of structured TAGs, the molecular position of n-3 PUFAs on TAG did not seem to play a critical role [26]. Whether this also applies for natural TAGs with complex fatty acid compositions remains to be elucidated, particularly in specific cells and tissues, not only in total plasma. Anyway, seal oil is an interesting alternative source of n-3 PUFAs.

### Duodenal administration of dietary oils

During gastric emptying only a few ml of gastric content is passed through to the duodenum per minute. Acute administration of 10 ml of oil directly into the duodenum is clearly unphysiological and might yield a higher bolus of n-3 PUFAs into the circulation as compared to oral administration [12]. However our unpublished data showed comparable plasma incorporation of n-3 PUFAs from seal oil (10 ml × 3 per day) during duodenal versus oral administration in healthy volunteers (*n* = 16), and a tendency to abnormally high faecal fat levels. Thus malabsorbed n-3 PUFAs may have passed to the colon where it can potentially reinforce the mucus as seen with retarded release phosphatidylcholine in UC [27,28]. Studies of n-3 PUFA administrations in IBD have showed inconsistent findings [29], possibly due to variations in study design. One of the studies showing most beneficial effect (reduced relapses in CD), applied fish oil capsules which contained free fatty acids, coated in order to resist gastric acid [30]. I.e. delivery to the distal gut may be important in order to achieve beneficial effects of n-3 PUFA administrations.

Recently, high fat enteral nutrition [31,32] was shown to activate a 'nicotinic anti-inflammatory pathway', mediated by the vagus nerve, being able to inhibit pro-inflammatory cytokine production [33,34]. Duodenal administration of seal oil to the principal site of lipid digestion yields free long chain n-3 PUFAs known to stimulate cholecystokinin (CCK) release [35]. CCK is an important neuro-transmitter of the afferent vagus nerve. Hence, duodenal administration of bolus doses of 10 ml seal oil three times daily may strongly activate the vago-vagal, anti-inflammatory reflex and partly explain the rapid amelioration of bodily pain. By similar mechanisms trauma and surgery patients may benefit from early enteral nutrition [32]. However, further studies of the effects of these forms of fat administrations are needed.

## Conclusion

In these two separate studies, short-term duodenal administration of seal oil normalised the n-6 to n-3 FA ratio in rectal mucosa and improved the bodily pain dimension of HRQOL of patients with IBD-related joint pain. The possibility of a causal relationship between n-6 to n-3 FA ratio in rectal mucosa and bodily pain in IBD patients warrants further investigations.

## Competing interests

The author(s) declare that they have no competing interests.

## Authors' contributions

TB contributed to the design of the study, coordinated and participated in the data collection, statistical analysis and drafted the manuscript. JGB contributed to the design of the study, collected data and helped with interpretation of data. MV, GA and RL collected data and helped with interpretation of data. LAB, AB and LF conceived of the study and designed the study, and LF in addition helped draft the manuscript. All authors read and approved the final manuscript.
